# Digital Mental Health Intervention Plus Usual Care Compared With Usual Care Only and Usual Care Plus In-Person Psychological Counseling for Orthopedic Patients With Symptoms of Depression or Anxiety: Cohort Study

**DOI:** 10.2196/36203

**Published:** 2022-05-04

**Authors:** Ashwin J Leo, Matthew J Schuelke, Devyani M Hunt, J Philip Miller, Patricia A Areán, Abby L Cheng

**Affiliations:** 1 Washington University in St Louis School of Medicine St Louis, MO United States; 2 Division of Biostatistics Washington University in St Louis School of Medicine St Louis, MO United States; 3 Division of Physical Medicine and Rehabilitation Department of Orthopaedic Surgery Washington University in St Louis School of Medicine St Louis, MO United States; 4 Department of Psychiatry and Behavioral Sciences University of Washington Seattle, WA United States

**Keywords:** digital health, mental health, depression, anxiety, chronic pain, musculoskeletal, orthopedic

## Abstract

**Background:**

Depression and anxiety frequently coexist with chronic musculoskeletal pain and can negatively impact patients’ responses to standard orthopedic treatments. Nevertheless, mental health is not routinely addressed in the orthopedic care setting. If effective, a digital mental health intervention may be a feasible and scalable method of addressing mental health in an orthopedic setting.

**Objective:**

We aimed to compare 2-month changes in mental and physical health between orthopedic patients who received a digital mental health intervention in addition to usual orthopedic care, those who received usual orthopedic care only (without a specific mental health intervention), and those who received in-person care with a psychologist as part of their orthopedic treatment plan.

**Methods:**

In this single-center retrospective cohort study involving ancillary analysis of a pilot feasibility study, 2-month self-reported health changes were compared between a cohort of orthopedic patients who received access to a digital mental health intervention (Wysa) and 2 convenience sample comparison cohorts (patients who received usual orthopedic care without a specific mental health intervention and patients who received in-person care with a psychologist as part of their orthopedic treatment plan). All patients were 18 years or older and reported elevated symptoms of depression or anxiety at an orthopedic clinic visit (Patient-Reported Outcomes Measurement Information System [PROMIS] Depression or Anxiety score ≥55). The digital intervention was a multi-component mobile app that used chatbot technology and text-based access to human counselors to provide cognitive behavioral therapy, mindfulness training, and sleep tools, among other features, with an emphasis on behavioral activation and pain acceptance. Outcomes of interest were between-cohort differences in the 2-month longitudinal changes in PROMIS Depression and Anxiety scores (primary outcomes) and PROMIS Pain Interference and Physical Function scores (secondary outcomes).

**Results:**

Among 153 patients (mean age 55, SD 15 years; 128 [83.7%] female; 51 patients per cohort), patients who received the digital mental health intervention showed clinically meaningful improvements at the 2-month follow-up for all PROMIS measures (mean longitudinal improvement 2.8-3.7 points; *P*≤.02). After controlling for age and BMI, the improvements in PROMIS Depression, Pain Interference, and Physical Function were meaningfully greater than longitudinal changes shown by patients who received usual orthopedic care (mean between-group difference 2.6-4.8 points; *P*≤.04). Improvements in PROMIS Physical Function were also meaningfully greater than longitudinal changes shown by patients who received in-person psychological counseling (mean between-group difference 2.4 points; *P*=.04).

**Conclusions:**

Patients who received a digital mental health intervention as part of orthopedic care reported greater 2-month mean improvements in depression, pain interference, and physical function than patients who received usual orthopedic care. They also reported a greater mean improvement in physical function and comparable improvements in depression, anxiety, and pain interference compared with orthopedic patients who received in-person psychological counseling.

## Introduction

### Background

Symptoms of depression and anxiety commonly coexist with chronic musculoskeletal pain. When this occurs, traditional mental health treatments are less effective in reducing psychological impairment, and traditional musculoskeletal treatments are less effective in addressing physical symptoms [1-5]. Specifically within the field of orthopedic care, pre-existing symptoms of depression or anxiety are associated with poor outcomes, such as worse postintervention physical functioning, increased postoperative opioid use, and reduced return-to-work rates [6-8]. Awareness of this phenomenon is growing, but mental health screening and intervention is still not considered a standard part of orthopedic care [9]. Barriers include orthopedic providers’ lack of time and available mental health resources to offer; patients’ financial resources, transportation, time, and stigma-related barriers to seeking mental health care, especially in person; and a national shortage of qualified mental health providers [9-11].

A digital mental health intervention is a promising tool to improve access to mental health care because it is not affected by many of the barriers that limit in-person care. That is, providers can easily refer patients to a digital resource because it is not limited by physical location or wait times, and patient challenges related to transportation, time, and fear of stigma are reduced and, in some cases, eliminated by enabling access to care without requiring travel to an in-person provider [12,13]. Furthermore, in a single-arm prospective pilot study, we demonstrated that delivery of a digital mental health intervention (Wysa) within an outpatient orthopedic care setting is feasible and preliminary effectiveness analyses are promising [14].

Outside the orthopedic setting, existing evidence supports the effectiveness of digital mental health interventions, but the effect size varies depending on the comparison arm, for instance, an inactive “usual care” or waitlist control group, or an active “gold standard” in-person counseling treatment group [15-17]. Therefore, the literature repeatedly calls for more comparison between digital mental health interventions and real-world treatment alternatives, both in the general population of people with symptoms of depression and anxiety and in those with coexisting symptoms of chronic pain [12,18-21]. To further understand the potential for introducing a digital mental health intervention within the context of orthopedic care, clinical improvements observed in our pilot study need to be compared to improvements made by comparable patients who received usual orthopedic care (without a dedicated mental health intervention) and by patients who received in-person mental health care as part of their orthopedic treatment plan. This added information would provide insight regarding whether a digital mental health intervention provides added clinical benefit compared with usual orthopedic care, and if so, whether that benefit is less than, equivalent to, or even superior to in-person mental health care that is offered in a similar setting.

### Objective

The purpose of this study was to compare 2-month changes in mental and physical health between orthopedic patients who received a digital mental health intervention (Wysa) in addition to usual orthopedic care, those who received usual orthopedic care only (without any specific mental health intervention), and those who received in-person care with a psychologist as part of their orthopedic treatment plan. We hypothesized that compared with patients who received usual care, orthopedic patients who were also provided a digital mental health intervention would report greater improvements in mental and physical health at a 2-month follow-up. Additionally, we hypothesized that compared with orthopedic patients who established in-person care with a psychologist, orthopedic patients who were provided a digital mental health intervention would report comparable improvements in mental and physical health at a 2-month follow-up.

## Methods

### Study Design

This was a retrospective study that involved ancillary analysis of data collected from a single-arm pilot feasibility study and other existing medical record data (ClinicalTrials.gov NCT04640090) [14]. All patients were evaluated within the orthopedic department of a single tertiary care academic medical center (Washington University) in the United States between 2017 and 2021.

### Ethics Approval

Washington University institutional review board approval was obtained prior to data collection (IRB #202005219).

### Participants

#### General Eligibility Criteria

All study participants were adults aged 18 years or older who presented to a nonoperative subspecialty-trained orthopedic provider for evaluation and management of musculoskeletal pain. As part of usual care, all patients who present to the orthopedic department of the study institution complete Patient-Reported Outcomes Measurement Information System (PROMIS) Depression and Anxiety measures prior to clinician evaluation. Only patients who self-reported elevated symptoms of depression or anxiety, as defined by scores of 55 or above on either measure or both measures, were eligible.

#### Digital Mental Health Intervention (Wysa) Cohort

The primary cohort of interest included patients who enrolled in a single-arm, prospective cohort, pilot feasibility study in which they received 2 months of complimentary access to a digital mental health intervention (Wysa), in addition to their usual orthopedic care. The intervention and this cohort have previously been described [14]. In brief, Wysa is a multi-component mobile app based on the principles of cognitive behavioral therapy, mindfulness, and motivational interviewing [22-24]. It includes an artificial intelligence–based “chatbot” conversational agent and text-based access to human “coach” counselors who have master’s degrees in psychology. A commercial version of the app exists, but for the pilot study, additional novel features were incorporated to specifically tailor the experience for people with chronic pain. Additional features were based on the principles of behavioral activation and pain acceptance [25-30]. These patients were recruited for the pilot study between December 8, 2020, and July 14, 2021. Patients who were planning to start in-person psychological treatment were excluded from the pilot study, and only 51 of 61 (84%) enrolled patients who completed 2-month PROMIS follow-up assessments were eligible for this ancillary analysis. While the majority of patients engaged with the intervention multiple times during the study period [14], lack of engagement with the intervention was not an exclusion criterion.

#### Usual Orthopedic Care Cohort

The Wysa cohort was compared to a “usual orthopedic care” cohort, which was a convenience sample of patients who presented to the same orthopedic clinics as those who enrolled in the prospective Wysa study but on days during which recruitment for the Wysa study was not occurring. In these clinics, “usual orthopedic care” most commonly includes physical therapy, medications (eg, nonsteroidal anti-inflammatory drugs, oral steroids, and neuropathic pain medications), and steroid injections, as appropriate. Other less commonly recommended procedures include radiofrequency ablation, manual massage, and acupuncture. These patients could have been presenting for a new or follow-up evaluation (just like in the Wysa cohort), but their orthopedic management plan could not have included any dedicated mental health management, such as counseling or a digital intervention. Furthermore, follow-up PROMIS Depression and Anxiety scores had to be documented in the medical record between 1 and 3 months after the initial evaluation. If scores from multiple dates were available, the scores closest to a 2-month follow-up duration were selected for analysis. Patients who presented for an acute injury or a procedure were excluded. Patients in this cohort were identified consecutively via a reverse chronological medical record review until a sample size of 51 patients (matching the Wysa cohort) was reached. As a result, the baseline clinic date for patients in this cohort spanned September 16, 2021, to December 10, 2021, with a mean follow-up time of 56 (SD 9) days.

#### In-Person Psychological Counseling (“Gold Standard”) Cohort

The Wysa cohort was also compared to a “gold standard” cohort that received in-person psychological counseling as part of the orthopedic treatment plan. This was also a convenience sample of patients who presented to the same orthopedic clinics as those who enrolled in the prospective Wysa study. However, as part of their orthopedic treatment plan, they initiated in-person psychological counseling with a licensed clinical psychologist having over 20 years of experience. The psychologist delivered cognitive behavioral therapy, motivational interviewing, and mindfulness and deep breathing training, as indicated, during treatment sessions. This unique service was available through a lifestyle medicine–based center within the study institution’s orthopedic department [31]. The center’s purpose is to help patients manage musculoskeletal conditions by addressing underlying lifestyle habits and biopsychosocial comorbidities that contribute to musculoskeletal pain. To be eligible for this study, patients in this cohort had to have completed their evaluation with the psychologist within 2 weeks of completing baseline PROMIS Depression and Anxiety measures, and they had to have completed at least one follow-up session with the psychologist prior to the follow-up date designated for this study. For patients with PROMIS scores at numerous time points, scores obtained closest to the initial psychology evaluation and to a 2-month follow-up duration were selected for analysis. Patients were identified consecutively via a reverse chronological medical record review until a sample size of 51 patients (matching the Wysa cohort) was reached. As a result, the baseline clinic date for patients in this cohort spanned August 21, 2017, to September 20, 2021, with a mean follow-up time point of 62 (SD 19) days later.

### Variables

As previously described, PROMIS scores from the Wysa cohort were collected prospectively during the pilot feasibility study. The rest of the data from all 3 cohorts were obtained from a medical record review of information collected as standard care during patients’ clinical encounters. All data extraction from patients’ medical records was performed by a single study team member (AJL). Patients’ self-reported mental and physical health was measured using the PROMIS Computer Adaptive Test (CAT) Adult Depression v1.0, Anxiety v1.0, Pain Interference v1.1, and Physical Function v2.0 measures [32-36]. PROMIS scores were normalized to the general US population, with a mean of 50 and SD of 10. Higher scores represent “more” of the domain [37]. For example, high scores on PROMIS Depression are unfavorable, but high scores on PROMIS Physical Function are favorable. Descriptive variables collected included demographics (age, sex, race, ethnicity, and Area Deprivation Index [38,39]), BMI, pain location and duration, and medical history (hypertension, hyperlipidemia, heart disease, lung disease, diabetes, sleep apnea, depression, and anxiety).

### Outcomes

The primary outcomes were the between-cohort differences in 2-month longitudinal change in PROMIS Depression and Anxiety scores. The secondary outcomes were the between-cohort differences in 2-month longitudinal change in PROMIS Pain Interference and Physical Function scores. Minimum clinically meaningful effect sizes were a priori set to match thresholds used in the pilot feasibility study, which were determined from previously published literature in conservatively managed orthopedic patients with chronic musculoskeletal pain. Minimum meaningful effect sizes were defined as at least 3.2 points on PROMIS Depression, 3.0 points on PROMIS Anxiety, 2.0 points on PROMIS Pain Interference, and 2.2 points on PROMIS Physical Function [40-42]. Between-cohort differences in baseline descriptive variables were examined, as well.

### Statistical Analysis

Univariate descriptive statistics per cohort were calculated for all baseline study variables. Differences between the “digital mental health intervention” cohort and each of the 2 comparison cohorts (“usual orthopedic care” and “in-person psychological counseling”) were calculated as either mean differences via Welch 2-sample *t* tests or percentage differences via 2-sample *t* tests for equality of proportions with Yates’ continuity correction. Average within-person changes in PROMIS scores in each cohort were calculated using paired *t* tests. Comparisons of the 2-month change in PROMIS scores between the “digital mental health intervention” cohort and each of the 2 comparison cohorts were assessed with linear mixed models. Mean longitudinal changes in health were estimated via slope coefficients, and comparisons of slopes were tested with time by cohort interaction terms. Initially, age and BMI (as a marker for metabolic health) were added to the models as covariates because these baseline characteristics were different between cohorts. However, these adjustments failed to alter the models in a statistically or clinically meaningful way, so unadjusted models are reported. Missing baseline descriptive data were omitted from relevant analyses. One participant in the “in-person psychological counseling” cohort was missing the follow-up PROMIS Depression score. Median imputation was performed for this single value. Significance was a priori set at *P*<.05. The sample size for each cohort was set to match the available sample size of the Wysa cohort. Data were collected using Research Electronic Data Capture (REDCap) [43,44], and statistical analyses were performed using R (v4.0.2, R Core Team).

## Results

### Patient Characteristics

Of the 153 patients included, the mean age was 55 (SD 15) years (range 18-86 years), and 128 (83.7%) were female. Compared with the cohort that received usual orthopedic care, patients who received the digital mental health intervention (Wysa) had a higher prevalence of sleep apnea (14/51, 27% vs 5/50, 10%; percent difference 18%, 95% CI 1%-34%; *P*=.05), but otherwise, had similar demographic, musculoskeletal, and medical characteristics (Table 1). Compared with the cohort that received in-person counseling, patients who received the digital mental health intervention were somewhat younger (mean age 53.2 vs 59.5 years; mean difference −6.4 years, 95% CI −12.0 to −0.4; *P*=.03), were less likely to have low back pain (28/51, 55% vs 42/51, 82%; percent difference −28%, 95% CI −47% to −8%; *P*=.006), had a lower BMI (indicative of obesity; mean 29.1 kg/m^2^ vs 38.1 kg/m^2^; mean difference −9.0, 95% CI −12.1 to −6.0; *P*<.001), and had a lower prevalence of both hypertension (21/50, 42% vs 36/47, 77%; percent difference −35%, 95% CI −55% to −14%; *P*=.001) and sleep apnea (14/51, 27% vs 28/50, 56%; percent difference −29%, 95% CI −49% to −8%; *P*=.007). There were no meaningful between-cohort differences in the proportion of patients who had a documented diagnosis of depression or anxiety.

**Table 1:** Sociodemographic and medical history characteristics in the 3 cohorts of patients.

Characteristic		Digital mental health intervention (n=51)	Usual orthopedic care (n=51)			In-person psychological counseling (n=51)		
		Value, mean (SD) or n/N (%)	Value, mean (SD) or n/N (%)	MD^a^ (95% CI) or % Diff^b^ (95% CI)^c^	*P* value^c^	Value, mean (SD) or n/N (%)	MD (95% CI) or % Diff (95% CI)^c^	*P* value^c^
Age (years)		53.2 (14.6)	52.7 (15.6)	−0.4 (−6.3 to 5.5)	.89	59.5 (14.1)	6.4 (0.4 to 12.0)	.03
**Sex**								
	Female	44/51 (86)	43/51 (84)	−2% (−18 to 14)	>.99	41/51 (80)	−6% (−22 to 11)	.60
	Male	7/51 (14)	8/51 (16)	2% (−14 to 18)	>.99	10/51 (20)	6% (−11 to 22)	.60
**Race**								
	White	46/51 (90)	43/51 (84)	−6% (−21 to 9)	.55	41/51 (80)	−10% (−25 to 6)	.26
	Black	5/51 (10)	6/51 (12)	2% (−12 to 16)	>.99	8/51 (16)	6% (−9 to 21)	.55
	Asian	0/51 (0)	1/51 (2)	2% (−4 to 8)	>.99	1/51 (2)	2% (−4 to 8)	>.99
	Other	0/51 (0)	1/51 (2)	2% (−4 to 8)	>.99	1/51 (2)	2% (−4 to 8)	>.99
**Ethnicity**								
	Hispanic	2/51 (4)	0/51 (0)	−4% (−11 to 3)	.48	1/51 (2)	−2% (−11 to 7)	>.99
	Not Hispanic	49/51 (96)	51/51 (100)	4% (−3 to 11)	.48	50/51 (98)	2% (−7 to 11)	>.99
**Area Deprivation Index^d^**								
	Quartile 1 (least deprived)	17/51 (33)	12/51 (24)	−10% (−29 to 10)	.38	18/51 (35)	2% (−18 to 22)	>.99
	Quartile 2	16/51 (31)	23/51 (45)	14% (−7 to 34)	.22	19/51 (37)	6% (−15 to 26)	.68
	Quartile 3	10/51 (20)	9/51 (18)	−2% (−19 to 15)	>.99	9/51 (18)	−2% (−19 to 15)	>.99
	Quartile 4 (most deprived)	8/51 (16)	7/51 (14)	−2% (−18 to 147)	>.99	5/51 (10)	−6% (−21 to 9)	.55
Pain duration (years)		6.8 (8.2)	8.1 (6.5)	1.4 (−1.6 to 4.3)	.36	8.4 (7.3)	1.6 (−1.5 to 4.7)	.30
**Pain location^e^**								
	Low back	28/51 (55)	31/51 (61)	6% (−15 to 27)	.69	42/51 (82)	28% (8 to 47)	.006
	Leg	38/51 (75)	37/51 (73)	−2% (−21 to 17)	>.99	43/51 (84)	10% (−8 to 27)	.33
	Neck	16/51 (31)	11/51 (22)	−10% (−29 to 9)	.37	9/51 (18)	−14% (−32 to 5)	.17
	Arm	14/51 (27)	10/51 (20)	−8% (−26 to 11)	.48	9/51 (18)	−10% (−28 to 8)	.34
	Generalized pain	5/51 (10)	4/51 (8)	−2% (−15 to 11)	>.99	7/51 (14)	4% (−11 to 18)	.76
BMI (kg/m^2^)		29.1 (7.2)	26.9 (6.4)	−2.3 (−4.9 to 0.4)	.10	38.1 (8.4)	9.0 (6.0 to 12.1)	<.001
**Medical history**								
	Hypertension	21/50 (42)	20/48 (42)	0% (−20 to 20)	>.99	36/47 (77)	35% (14 to 55)	.001
	Hyperlipidemia	31/49 (63)	30/48 (64)	−1% (−21 to 19)	>.99	36/46 (78)	15% (−5 to 35)	.17
	Cardiovascular disease	7/50 (14)	8/50 (16)	2% (−14 to 18)	>.99	9/50 (18)	4% (−12 to 20)	.79
	Lung disease	5/51 (10)	4/51 (8)	−2% (−15 to 11)	>.99	6/50 (12)	2% (−12 to 16)	.97
	Diabetes	5/48 (10)	6/47 (13)	2% (−13 to 17)	.97	13/46 (28)	18% (0 to 36)	.053
	Sleep apnea	14/51 (27)	5/50 (10)	−18% (−34 to −1)	.05	28/50 (56)	29% (8 to 49)	.007
	Depression	33/50 (66)	33/50 (66)	0% (−19 to 19)	>.99	37/49 (76)	10% (−10 to 230)	.41
	Anxiety	36/50 (72)	38/49 (78)	6% (−14 to 25)	.69	36/46 (78)	6% (−13 to 26)	.64

^a^MD: mean difference.

^b^% Diff: percent difference.

^c^All bivariate analyses involve comparisons with the “digital mental health intervention” group.

^d^The national Area Deprivation Index is a neighborhood-level measure of social disadvantage based on a person’s US Census Block Group [38,39].

^e^Some patients reported multiple pain locations.

### Primary Outcomes: Mental Health

**Figure 1:**
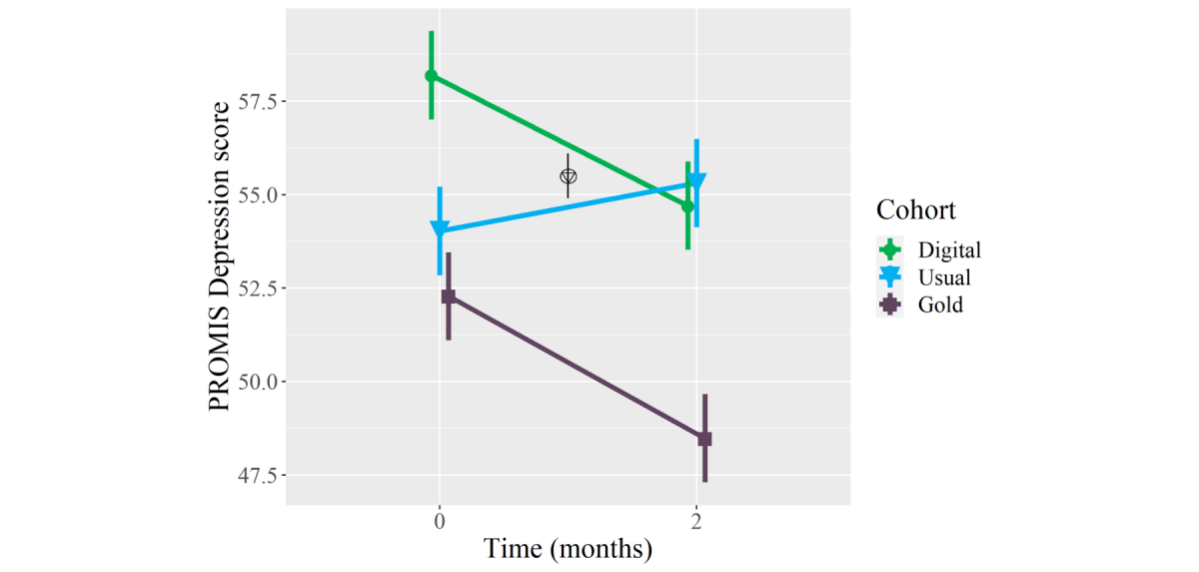
Mean longitudinal change in Patient-Reported Outcomes Measurement Information System (PROMIS) Depression scores over a 2-month follow-up in patients who, as part of orthopedic care, were provided a digital mental health intervention (Wysa) (n=51) (green circles), received usual orthopedic care (n=51) (blue triangles), or received “gold standard” in-person care with a psychologist (n=51) (purple squares). The triangle within a circle signifies a between-cohort difference in the longitudinal change between the digital mental health intervention cohort and usual orthopedic care cohort. Error bars represent standard error.

**Figure 2:**
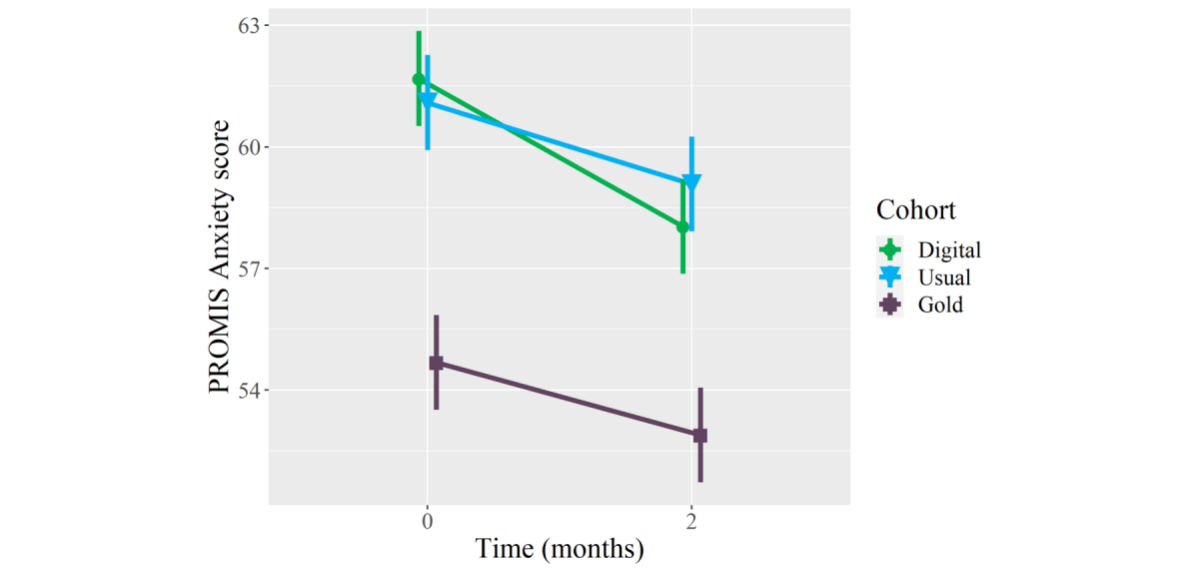
Mean longitudinal change in Patient-Reported Outcomes Measurement Information System (PROMIS) Anxiety scores over a 2-month follow-up in patients who, as part of orthopedic care, were provided a digital mental health intervention (Wysa) (n=51) (green circles), received usual orthopedic care (n=51) (blue triangles), or received “gold standard” in-person care with a psychologist (n=51) (purple squares). Error bars represent standard error.

On average, patients who received the digital mental health intervention showed clinically meaningful improvements in PROMIS Depression (mean longitudinal change −3.5 points, 95% CI −5.9 to −1.1; *P*=.006) and Anxiety (−3.7 points, 95% CI −5.9 to −1.4; *P*=.002) scores at the 2-month follow-up, whereas patients who received usual orthopedic care did not show clinically meaningful improvements in these measures, and patients who received in-person psychological counseling only showed meaningful improvements in PROMIS Depression scores (−3.8 points, 95% CI −5.9 to −1.6; *P*=.001) (Table 2). Furthermore, patients who received the digital mental health intervention showed meaningfully greater mean improvements in PROMIS Depression (but not Anxiety) scores than patients who received usual orthopedic care (mean between-group difference −4.8 points, 95% CI −7.6 to −1.9; *P*=.001), and there were no statistically significant or clinically meaningful between-group differences in longitudinal improvements in PROMIS Depression or Anxiety scores between patients who received the digital mental health intervention and those who received in-person psychological counseling (Table 3; Figures 1 and 2). Adjusting for age and BMI did not have a statistically or clinically meaningful effect on these results.

**Table 2:** Mental and physical health changes (measured by the Patient-Reported Outcomes Measurement Information System) across a 2-month follow-up in the 3 cohorts of patients (n=51 for each patient cohort).

PROMIS^a^ domain^b^		Baseline score, mean (SE)	2-month follow-up score, mean (SE)	Within-group longitudinal change, mean (95% CI)	*P* value
**Depression**					
	Digital mental health intervention	58.2 (6.8)	54.7 (8.7)	−3.5 (−5.9 to −1.1)	.006
	Usual orthopedic care	54.0 (7.1)	55.3 (6.6)	1.3 (−2.9 to 0.4)	.12
	In-person psychological counseling	52.3 (9.9)	48.4 (10.7)	−3.8 (−5.9 to −1.7)	.001
**Anxiety**					
	Digital mental health intervention	61.7 (5.8)	58.0 (7.8)	−3.7 (−5.9 to −1.4)	.002
	Usual orthopedic care	61.1 (5.8)	59.1 (7.0)	−2.0 (−3.6 to −0.4)	.02
	In-person psychological counseling	54.7 (10.4)	52.9 (11.5)	−1.8 (−3.7 to 0.1)	.06
**Pain Interference**					
	Digital mental health intervention	64.9 (6.4)	62.1 (7.0)	−2.8 (−5.2 to −0.4)	.02
	Usual orthopedic care	66.0 (5.2)	65.8 (5.0)	−0.2 (−1.4 to 1.1)	.77
	In-person psychological counseling	64.7 (6.4)	63.1 (6.4)	−1.6 (−3.0 to −0.2)	.03
**Physical Function**					
	Digital mental health intervention	36.1 (6.5)	39.5 (6.7)	3.3 (1.3 to 5.4)	.002
	Usual orthopedic care	35.1 (6.9)	35.7 (6.6)	0.6 (−1.0 to 2.3)	.45
	In-person psychological counseling	34.1 (6.1)	35.0 (5.9)	1.0 (−0.1 to 2.0)	.08

^a^PROMIS: Patient-Reported Outcomes Measurement Information System.

^b^Higher scores on PROMIS Depression, Anxiety, and Pain Interference indicate worse symptoms. Higher scores on PROMIS Physical Function indicate better function. Clinically meaningful effect sizes are defined as at least 3.2 points for PROMIS Depression, 3.0 points for Anxiety, 2.0 points for Pain Interference, and 2.2 points for Physical Function [40-42].

**Table 3:** Between-group differences in 2-month mental and physical health symptom changes in the 3 cohorts of patients.

PROMIS^a^ domain^b^	Digital mental health intervention (n=51)	Usual orthopedic care (n=51)				In-person psychological counseling (n=51)			
	Mean longitudinal change	Mean longitudinal change	Mean between-group difference^c^	95% CI^c^	*P* value^c^	Mean longitudinal change	Mean between-group difference^c^	95% CI^c^	*P* value^c^
Depression^d^	−3.5	1.3	−4.8	−7.6 to −1.9	.001	−3.8	0.3	−2.6 to 3.2	.83
Anxiety	−3.7	−2.0	−1.6	−4.3 to 1.1	.23	−1.8	−1.9	−4.5 to 0.8	.18
Pain Interference	−2.8	−0.2	−2.6	−5.1 to −0.2	.04	−1.6	−1.2	−3.6 to 1.2	.34
Physical Function	3.3	0.6	2.7	0.5 to 5.0	.02	1.0	2.4	0.2 to 4.7	.04

^a^PROMIS: Patient-Reported Outcomes Measurement Information System.

^b^Clinically meaningful effect sizes are defined as at least 3.2 points for PROMIS Depression, 3.0 points for Anxiety, 2.0 points for Pain Interference, and 2.2 points for Physical Function [40-42].

^c^All bivariate analyses involve comparisons with the “digital mental health intervention” group.

^d^One participant in the “in-person psychological counseling” cohort was missing the follow-up PROMIS Depression score. Median imputation was performed for this single value.

### Secondary Outcomes: Physical Health

**Figure 3:**
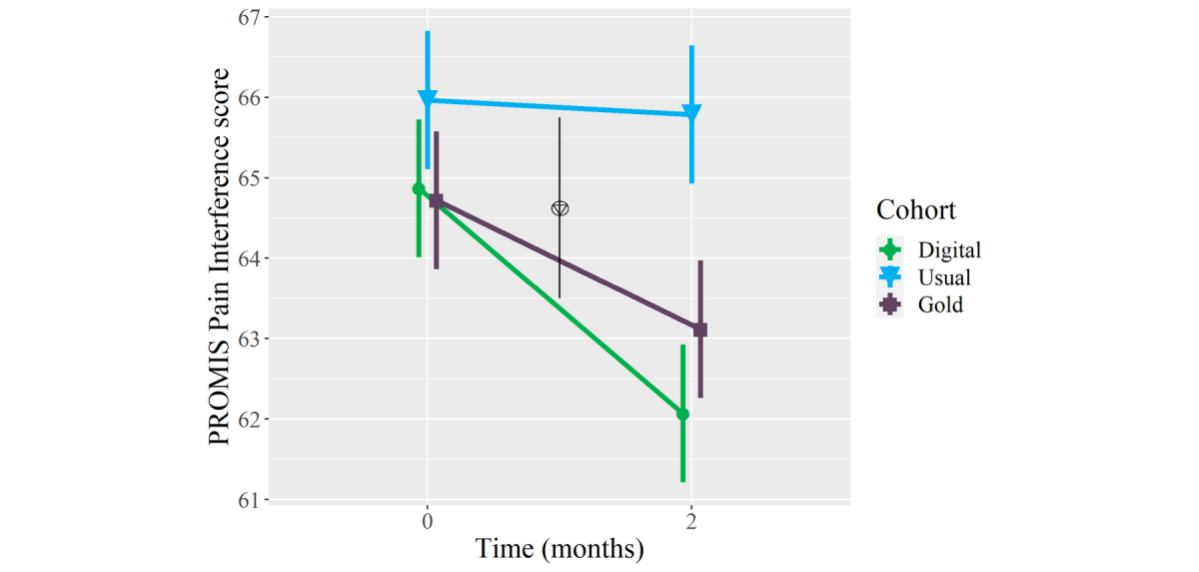
Mean longitudinal change in Patient-Reported Outcomes Measurement Information System (PROMIS) Pain Interference scores over a 2-month follow-up in orthopedic patients who, as part of orthopedic care, were provided a digital mental health intervention (Wysa) (n=51) (green circles), received usual orthopedic care (n=51) (blue triangles), or received “gold standard” in-person care with a psychologist (n=51) (purple squares). The triangle within a circle signifies a between-cohort difference in the longitudinal change between the digital mental health intervention cohort and usual orthopedic care cohort. Error bars represent standard error.

**Figure 4:**
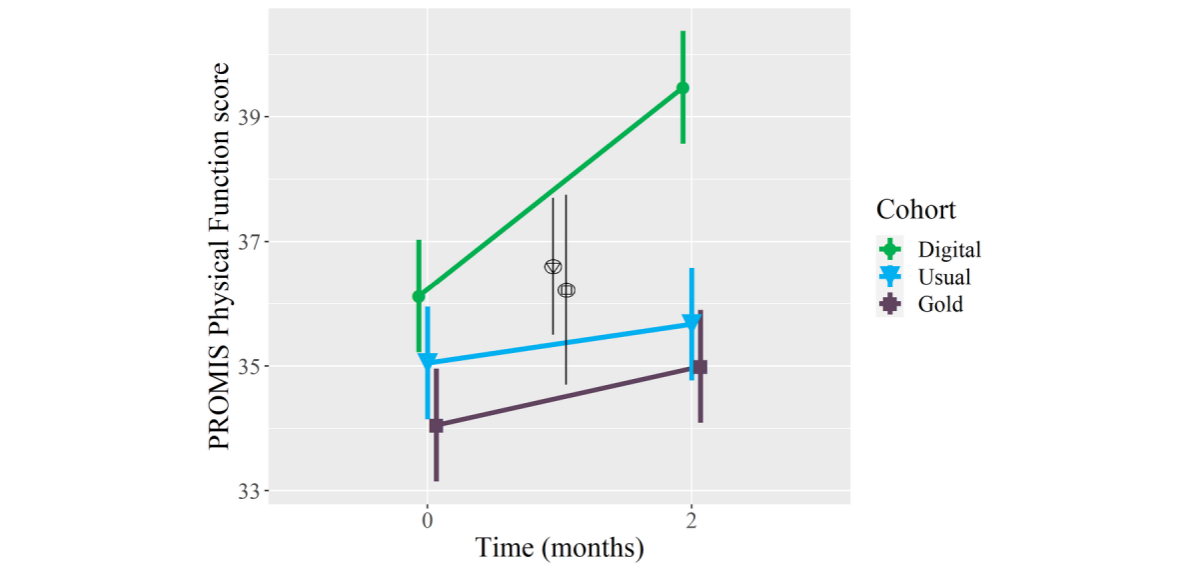
Mean longitudinal change in Patient-Reported Outcomes Measurement Information System (PROMIS) Physical Function scores over a 2-month follow-up in orthopedic patients who, as part of orthopedic care, were provided a digital mental health intervention (Wysa) (n=51) (green circles), received usual orthopedic care (n=51) (blue triangles), or received “gold standard” in-person care with a psychologist (n=51) (purple squares). The triangle within a circle signifies a between-cohort difference in the longitudinal change between the digital mental health intervention cohort and usual orthopedic care cohort. The square within a circle signifies a between-cohort difference in the longitudinal change between the digital mental health intervention cohort and “gold standard” in-person psychological counseling cohort. Error bars represent standard error.

On average, patients who received the digital mental health intervention showed clinically meaningful improvements in PROMIS Pain Interference (mean longitudinal change −2.8 points, 95% CI −5.2 to −0.4; *P*=.02) and Physical Function (3.3 points, 95% CI 1.3 to 5.4; *P*=.002) scores at the 2-month follow-up, whereas patients who received usual orthopedic care or in-person psychological counseling in conjunction with usual orthopedic care did not show meaningful improvements in these measures (Table 2). Furthermore, patients who received the digital mental health intervention showed meaningfully greater mean improvements in PROMIS Pain Interference scores than patients who received usual orthopedic care (mean between-group difference −2.6 points, 95% CI −5.1 to −0.2; *P*=.04), and they showed meaningfully greater mean improvements in PROMIS Physical Function scores than patients in either comparison cohort (mean between-group differences 2.4-2.7 points; *P*=.02 to .04) (Table 3; Figures 3 and 4). Adjusting for age and BMI did not have a statistically or clinically meaningful effect on these results.

## Discussion

### Principal Findings

To understand the potential benefit of introducing a digital mental health intervention in the context of orthopedic care for patients with coexisting symptoms of depression or anxiety, it is necessary to understand (1) the added clinical benefit of this intervention compared with usual orthopedic care, and (2) the clinical benefit of this intervention relative to the benefit achieved via usual orthopedic care supplemented by in-person counseling with a psychologist. In this retrospective cohort study, compared with patients who received usual orthopedic care, patients who also received a digital mental health intervention reported meaningfully greater 2-month mean improvements in depression (mean PROMIS between-group difference −4.8 points, 95% CI −7.6 to −1.9; *P*=.001), pain interference (−2.6 points, 95% CI −5.1 to −0.2; *P*=.04), and physical function (2.7 points, 95% CI 0.5 to 5.0; *P*=.02). Compared with patients who initiated in-person psychological counseling as part of their orthopedic treatment plan, patients who received a digital mental health intervention reported a meaningfully greater mean improvement in physical function (2.4 points, 95% CI 0.2 to 4.7; *P*=.04) and comparable improvements in depression, anxiety, and pain interference. These between-group differences were present even after controlling for baseline between-group differences in age and BMI.

### Strengths and Limitations

This study adds clinical context to our previously reported finding that it is feasible to deliver a digital mental health intervention in the setting of orthopedic care [14]. In other words, the primary strength of this study is the comparison of outcomes between orthopedic patients who received a digital mental health intervention, a “usual care” cohort, and a “gold standard” in-person psychological counseling cohort, especially because psychological counseling is still rarely prescribed in the context of orthopedic care.

The primary study limitations relate to the retrospective design and between-cohort baseline differences. Nonrandomized study designs have inherent limitations that can affect results. These include possible selection bias, differential attrition, regression to the mean, effects by unmeasured confounding variables (eg, concomitant orthopedic and mental health interventions), potential undetected interaction effects, and temporal/historical bias. Because patients who received the digital mental health intervention were actively enrolled into the research study while comparison cohorts were retrospectively selected as convenience samples, the digital intervention cohort could have been subject to healthy participant bias (in which increased activation in their health care could have contributed to greater health-related improvements). Furthermore, only 51 of 61 participants who completed 2-month follow-up measures in the digital intervention prospective study could be included in this longitudinal analysis, which also could have contributed to healthy participant bias. Additionally, the “usual orthopedic care” cohort could have been biased toward patients who were not showing satisfactory improvements and therefore returned to the clinic for a follow-up visit to determine the next steps. Similarly, it is possible that patients who received in-person psychological counseling as part of their orthopedic treatment plan had more “treatment-resistant” symptoms than patients in the other cohorts because in our experience, patients who choose this biopsychosocial lifestyle medicine approach to orthopedic care report feeling “at the end of their rope” and have often already tried many standard orthopedic treatments. A difference-in-differences quasiexperimental design was adopted, rather than propensity score matching, so that baseline between-group differences could be explored and because the number of potentially eligible patients for the in-person psychological counseling cohort was limited. Nevertheless, the vast majority of baseline characteristics were not significantly different between the cohorts, and baseline PROMIS scores for the digital intervention cohort were comparable (and at times worse) than the comparison cohorts, which would suggest that the digital intervention cohort was not experiencing less severe symptoms at baseline. While worse baseline scores provide more “room to improve,” the digital intervention cohort did not have worse baseline scores for any of the between-group differences that were found to be statistically significant.

Finally, generalizability is a limitation of this single-center study conducted at a tertiary care center. In order for a digital mental health intervention to be feasibly offered in an orthopedic setting, clinical providers need to support the initiative, and mental health screening needs to be a standard part of a patient’s evaluation, as is the case at the study institution.

### Comparison With Prior Work

Our findings are consistent with previous large randomized controlled trials and meta-analyses that have demonstrated the effectiveness of digital interventions for improving symptoms of depression, anxiety, and even pain-related impairment in people with chronic pain who have been recruited from pain management clinics, primary care clinics, and community/internet referral sources [20,21,45-47]. To our knowledge, though, this is the first study to specifically evaluate the effectiveness of a digital mental health intervention that was introduced in the setting of an orthopedic clinic, which is not typically a setting where mental health is a focus. We find it encouraging that, similar to studies of participants among the general population, orthopedic patients who received a digital mental health intervention reported favorable mental and physical health outcomes compared with those who received no active mental health treatment and at least comparable outcomes to those who initiated in-person care with a psychologist [15-17,48]. Our results add to the body of literature showing that the *impact* of pain on a person’s daily functioning and quality of life (ie, pain interference) can be improved by cognitive behavioral techniques, such as cognitive restructuring and addressing maladaptive thought patterns, regardless of the person’s level of physical function [49,50]. However, it was somewhat surprising that patients in this study who received usual orthopedic care did not make meaningful improvements in physical health. This could be related to (1) the inherent characteristics of nonoperative orthopedic patients who have multiple orthopedic clinic visits within a span of 2 months (eg, to address persistently bothersome symptoms), and (2) the difficulty of treating chronic pain (many years), especially when the interplay between mental health and chronic pain has not been sufficiently addressed. Nevertheless, it is encouraging that patients who received the digital mental health intervention made physical, as well as mental, health improvements even when the usual orthopedic care cohort did not.

### Directions for Future Study

Further investigation regarding the effectiveness of incorporating a digital mental health intervention into orthopedic care should include (1) a longer follow-up duration because depression and anxiety are chronic conditions; (2) dedicated evaluation of the intervention’s impact on sleep because pain and insomnia reciprocally affect each other and because digital cognitive behavioral interventions can be effective for insomnia [51-53]; and (3) more detailed information regarding concomitant orthopedic and mental health interventions, which are pursued during the intervention period. To obtain further insight, a fully powered, prospective, randomized controlled trial is needed.

### Conclusions

Patients who received a digital mental health intervention as part of their orthopedic care reported greater 2-month mean improvements in depression, pain interference, and physical function than patients who received usual orthopedic care without any specific mental health intervention. They also reported a greater mean improvement in physical function and comparable improvements in depression, anxiety, and pain interference compared with patients who initiated in-person psychological counseling as part of their orthopedic treatment plan. These differences met clinically meaningful thresholds and suggest that when orthopedic patients endorse elevated symptoms of depression or anxiety, incorporation of a digital mental health intervention into orthopedic care may improve patients’ physical and mental health outcomes relative to standard orthopedic care. Furthermore, improvements may be comparable to those achieved on incorporation of in-person psychological counseling. These retrospective findings warrant further investigation using a prospective randomized design.

## Abbreviations

PROMIS: Patient-Reported Outcomes Measurement Information System
